# Plasma-Derived Extracellular Vesicles Reveal Galectin-3 Binding Protein as Potential Biomarker for Early Detection of Glioma

**DOI:** 10.3389/fonc.2021.778754

**Published:** 2021-11-26

**Authors:** Rashmi Rana, Kirti Chauhan, Poonam Gautam, Mahesh Kulkarni, Reema Banarjee, Parul Chugh, Satnam Singh Chhabra, Rajesh Acharya, Samir Kumar Kalra, Anshul Gupta, Sunila Jain, Nirmal Kumar Ganguly

**Affiliations:** ^1^ Department of Research, Sir Ganga Ram Hospital, New Delhi, India; ^2^ Laboratory of Molecular Oncology, National Institute of Pathology, Indian Council of Medical Research (ICMR), New Delhi, India; ^3^ Biochemical Sciences Division, National Chemical Laboratory, Council of Scientific and Industrial Research (CSIR), Pune, India; ^4^ Department of Neurosurgery, Sir Ganga Ram Hospital, New Delhi, India; ^5^ Department of Histopathology, Sir Ganga Ram Hospital, New Delhi, India

**Keywords:** plasma-derived extracellular vesicles, galectin-3 binding protein, early detection, blood-based biomarker, proteomics

## Abstract

Gliomas are the most common type of the malignant brain tumor, which arise from glial cells. They make up about 40% of all primary brain tumors and around 70% of all primary malignant brain tumors. They can occur anywhere in the central nervous system (CNS) and have a poor prognosis. The average survival of glioma patients is approximately 6–15 months with poor aspects of life. In this edge, identification of proteins secreted by cancer cells is of special interest because it may provide a better understanding of tumor progression and provide early diagnosis of the diseases. Extracellular vesicles (EVs) were isolated from pooled plasma of healthy controls (n=03) and patients with different grades of glioma (Grade I or II or III, n=03 each). Nanoparticle tracking analysis, western blot, and flow cytometry were performed to determine the size, morphology, the concentration of glioma-derived vesicles and EV marker, CD63. Further, iTRAQ-based LC-MS/MS analysis of EV protein was performed to determine the differential protein abundance in extracellular vesicles across different glioma grades. We further verified galectin-3 binding protein (LGALS3BP) by ELISA in individual blood plasma and plasma-derived vesicles from control and glioma patients (n=40 each). Analysis by Max Quant identified 123 proteins from the pooled patient exosomes, out of which 34, 21, and 14 proteins were found to be differentially abundant by more than 1.3-fold in the different grades of glioma grade I, pilocytic astrocytoma; grade II, diffuse astrocytoma; grade III, anaplastic astrocytoma, respectively, in comparison with the control samples. A total of seven proteins—namely, CRP, SAA2, SERPINA3, SAA1, C4A, LV211, and KV112—showed differential abundance in all the three grades. LGALS3BP was seen to be upregulated across the different grades, and ELISA analysis from individual blood plasma and plasma-derived extracellular vesicles confirmed the increased expression of LGALS3BP in glioma patients (p<0.001). The present study provides LGALS3BP as a potential biomarker for early detection of glioma and improve survival outcome of the patient. The present study further provides the information of progression and monitoring the tumor grades (grade 1, grade II, grade III).

## Introduction

Gliomas are the most common type of malignant brain tumors that arise from glial cells. They make up about 40% of all primary brain tumors and around 70% of all primary malignant brain tumors (1). They can occur anywhere in central nervous system (CNS) and have poor prognosis. The average survival of glioma patients is approximately 7–15 months with poor aspects of life despite having undergone hostile surgical incisions and combined chemoradiation procedures. According to the 2016 WHO classification, the grading system have been made for the gliomas and differentiate them into four distinct grades, viz., grade I, pilocytic astrocytoma; grade II, diffuse astrocytoma; grade III, anaplastic astrocytoma; and grade IV, glioblastoma multiforme (2, 3). The surgical resection is the only way to resect tumor. Studies on glioma over the past decades have identified numerous factors that contribute to tumor progression and correlate to overall outcome such as patient’s age, grade, histopathology, and genetic abnormalities. Glioma cells proliferate and expand to distant organ sites by forming close interactions between tumor cells and the surrounding microenvironment (3). The tumor microenvironment evolves to accommodate the growing tumor (4, 5), but the molecular mechanisms involved are not well defined. Consequently, identification of proteins secreted by the cancer cells is of special interest because it may provide a better understanding of tumor progression and also provide early biomarkers of the disease.

In this context secreted proteins constitute an important class of molecules encoded by approximately 10% of the human genome (6). The term “secretome” was initially used to describe the study of proteins secreted by cells, tissues, or organisms that control many biological and physiological processes (7). More recently, the definition of secretome has been broadened to include the proteins released by both classical secretion mechanisms and those secreted in vesicles such as extracellular vesicles (8). The term “extracellular vesicles” was used for the first time by Trams et al. in 1981 and originated from “exfoliated membrane vesicles” with ectoenzyme activity, secreted by normal and neoplastic cells (9). Extracellular vesicles are nanometer-sized membrane vesicles (approximately 30–150 nm) with cup-shape morphology and contain various bioactive molecules including proteins, miRNA, mRNA, DNA, and lipids. Extracellular vesicles are formed by inward budding of the limiting membrane into the lumen of late endosomes (which are called multivesicular bodies or MVBs due to their multivesicular appearance) and then released into the extracellular milieu upon fusion with plasma membrane (10, 11). Extracellular vesicles are released by both normal and neoplastic cells and are present in several biological fluids such as blood plasma and urine, and considered to be an excellent resource for studying disease progression.

Recent studies suggest that vesicle shedding by malignant cells promotes tumor progression by stimulating neo-angiogenesis (12), carrying matrix remodeling enzymes (13), contributing to the preparation of the metastatic functions (14), and inducing antibody sequestration or drug resistance by drug exportation (15, 16). In the recent years, tumor-derived extracellular vesicles isolated from several body fluids have been proposed as relevant biological sources of prognostic biomarkers obtained in a non-invasive mode (17–19). In this study we have performed the first characterization and proteomic profile of tumor-derived extracellular vesicles from human glioma (grade I, II, III) to expand latest information on the molecular mechanisms of cancer progression and to propose potential tumor biomarkers. We found galectin-3 binding protein (LGALS3BP) as novel circulating biomarker for the detection of glioma at early grade and provide the information of progression in tumor grades.

## Materials and Methods

### Sample Collection and Characterization

**Figure 1:**
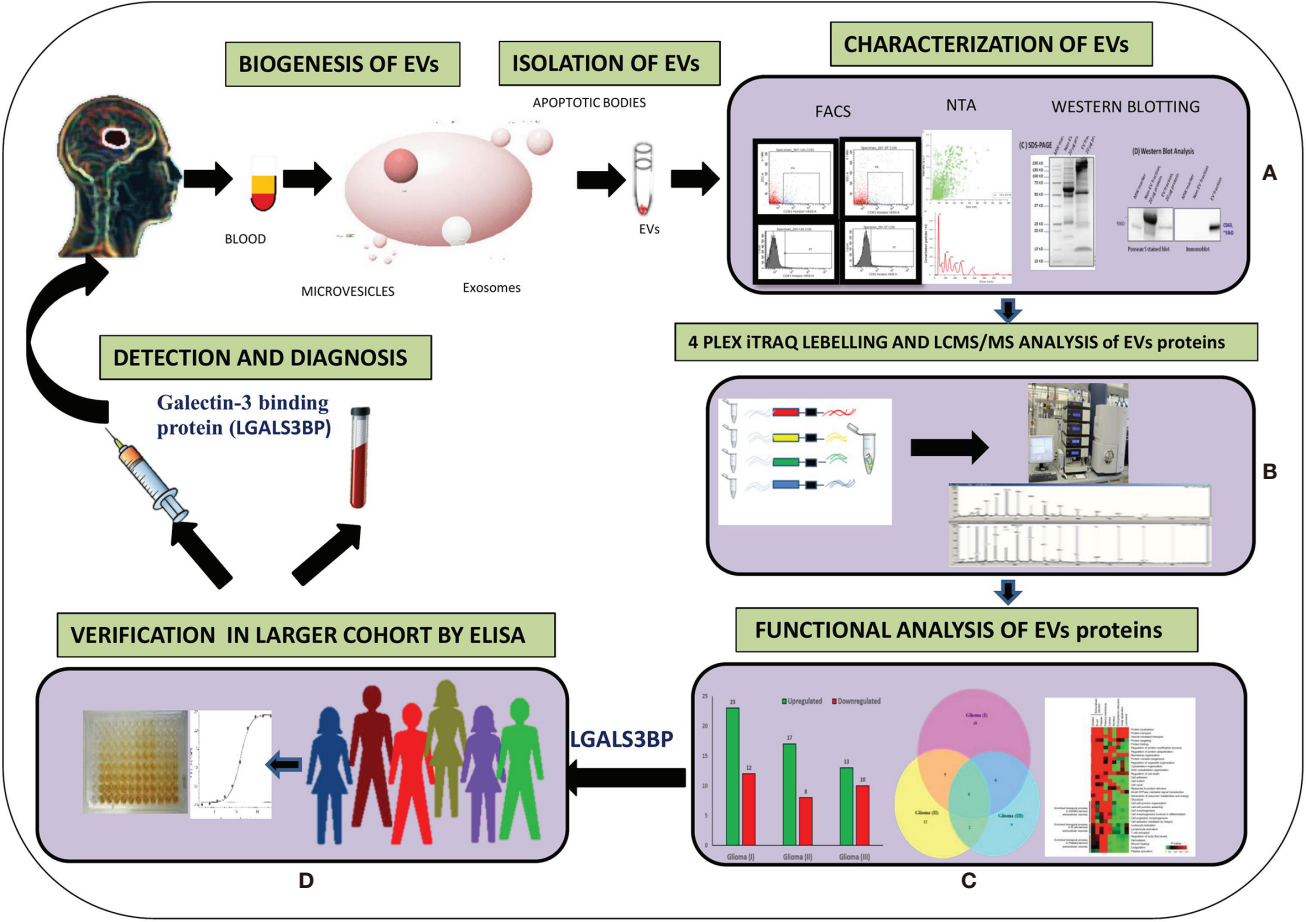
Graphical representation of methodology. **(A)** Extraction of EVs from plasma and characterization and quantification of EVs from different techniques. **(B)** Isobaric tag for relative and absolute quantitation (ITRAQ) labeling and Liquid Chromatography with tandem mass spectrometry (LC-MS/MS) analysis of EVs proteins. **(C)** Functional analysis of EVs altered proteins in different grades of Glioma. **(D)** Verification by Enzyme-linked immunosorbent assay (ELISA).

Blood samples (5 ml) were collected from three patients each from grade I, II, and III of glioma and three controls (Healthy individuals). We aliquoted 1.5 ml pooled plasma of each grade of glioma patients, and 1.5 ml pooled plasma of controls were taken for extracellular vesicles protein isolation for further analysis. These were the recently diagnosed patients whose clinical data and investigations were obtained from the clinicopathological referral sheets. Informed consent was obtained from each patient, and protocols were approved by the Sir Ganga Ram Hospital, Human Ethical Committee (Ref no. EC/10/17/1270), Delhi, India. All experiments were carried out in accordance with relevant guidelines and regulations. Glioma patients’ age was 46.38 ± 11.27 and controls age was 30.85 ± 5.15 for this study, respectively. Blood was allowed to settle at room temperature for 30 min. Whole blood was then centrifuged (Heraeus Megafuge 40R, Thermo Scientific) at 400 × *g* for 10 min at 20°C to remove cells. The plasma was collected and centrifuged again at 5,000 × *g* for 10 min at 20°C. The resulting platelet-poor plasma (PPP) was aliquoted and stored at −80°C. Clinical and histopathological evaluation of the tumor resections from the same patients was performed as per WHO guidelines for diagnosis. The histological diagnosis was made by the standard light-microscopic evaluation of sections stained with hematoxylin and eosin. Blood samples from healthy individuals, having no history of cancer, were collected and used as control. Graphical representation of overall methodology has been shown (**Figure 1**).

### Isolation and Characterization of EVs From Blood Plasma Samples

Extracellular vesicles were isolated from blood plasma using differential centrifugation technique (20). Briefly, blood plasma was diluted 1:4 with 1X PBS and centrifuged at 500 ×g at 4°C for 30 min to remove any cells. The supernatant was further centrifuged at 12,000 ×g at 4°C for 45 min to sediment any larger vesicles. Supernatant was filtered through a 0.22 µm PVDF MC membrane filter (Millipore, Manchester, USA) by centrifugation at 12,000 ×g for 2 min to remove vesicles larger than 220 nm. Exosomes were pelleted by ultracentrifugation at 1,20,000 ×g at 4°C for 1.5 h. EXO pellet was washed in PBS by centrifugation at 1,20,000 ×g at 4°C for 1.5 h and split into four parts for further studies. One part of the pellet was diluted with 50 µl 1X PBS for characterization by nanoparticle tracking analysis (NTA) for size and concentration. The second part of the pellet was mixed with denaturing dye and used for protein detection by SDS-PAGE and western blot analysis to study enrichment of exosomal markers, CD63. The third part was used to study CD63 level in different grades of glioma through FACS. Simultaneously, final and equal amount of pellet was used for mass spec analysis. The procedure was repeated three times for each experiment.

### Nanoparticle-Tracking Analysis

Extracellular vesicles were quantified *via* Brownian diffusion size analyses using Zeta View Nanoparticle-tracking analysis (NTA) instrument (Particle Metrix, Meerbusch, Germany). Sample aliquots were diluted 100-fold to achieve optimal concentration for analysis, and 1 ml of diluted sample was used for each analysis. Light scattering of individual particles in solution was digitally recorded, particle trajectory and displacement were automatically analyzed by image analysis tracking software, and the particle-size distribution was determined from the observed Brownian motion of individual particles according to the Stokes-Einstein relationship.

### Western Blot Analysis

Protein quantification was performed using Pierce BCA Protein Assay (Thermo Fisher Scientific, Rockford, IL, USA) according to the manufacturer’s recommended microplate assay procedure. Absorbance was measured with a Spectra Max M5 multimode microplate reader using Soft Max Pro data acquisition and analysis software (Molecular Devices, Sunnyvale, CA, USA). Vesicle isolates were denatured in 4X Laemmli sample buffer at 95°C for 10 min. Proteins were separated using 12% sodium dodecyl sulfate polyacrylamide gel electrophoresis in Tris/Glycine/SDS running buffer and transferred to Immun-Blot PVDF membrane (all reagents and supplies from Bio-Rad, Hercules, CA, USA). Immunoblotting was performed with the anti-CD63 antibody (Invitrogen by Life technologies, Carlsbad, CA, USA). Primary antibody incubation time was 16 h at 4°C (overnight). Washing was done with PBST for three times, and further blots were washed with PBS and incubated with appropriate HRP-conjugated secondary antibody for 2 h at 4°C (A9917, Sigma-Aldrich, MO, USA). The 3, 3’ Diaminobenzidine (DAB) was used for developing the membrane. Densitometry analysis was performed on digitized images using Image J image processing and analysis software.

### Flow Cytometry Analysis

Extracellular vesicles pellet 50 μl were dissolved with BCB buffer (PBS supplemented with 0.1% BSA; Roche, and 0.01% NaN_3_; G-Biosciences) for FACS preparation. Then 1 ml of BCB was added, and the sample was incubated overnight on rotation. Extracellular vesicles were pelleted by centrifugation at 2,000 ×g for 10 min, washed with 1 ml of BCB, and centrifuged again. The pellet was resuspended in 50 μl of BCB per analysis and stained using anti-Human CD9 (BV510), anti-Human CD63 (V450), anti-Human CD81 (PE) conjugated antibodies (BD Pharmingen™) for 30 min at 4°C. Negative control was obtained by incubating the diluted extracellular vesicles sample in the absence of primary antibody. Washing step was performed once after antibody labeling with 150 μl of BCB followed by centrifugation at 2,000 ×g for 10 min. Data were acquired in conventional flow cytometer (FACS Aria BD Biosciences, San Jose, CA, USA) and analyzed with the Flow Jo software (FACS Diva, version 6.1). One hundred thousand events were recorded at flow rate 1.0 event per second. All antibodies were used in accordance with manufacturer instructions.

### iTRAQ Labeling

Labeling of samples with iTRAQ reagents was carried out according to the manufacturer’s instructions (iTRAQ Reagents Multiplex kit; Applied Biosystems/MDS Sciex, Foster City, CA, USA). Briefly, 30 µg of pooled control or glioma (grade I, II, III) EV protein samples were reconstituted in dissolution buffer, denatured, reduced, alkylated, and then trypsinized (10 µg modified sequencing grade trypsin; Promega, Madison, WI, USA) for 16 h at 37°C. Tryptic digests were labeled with four different iTRAQ reagents. Control samples were labeled with 114, while the three grades of glioma samples with 115, 116, and 117 iTRAQ reagents, respectively. Reactions were quenched with glycine (10 mM).

### LC-MS/MS Analysis

Nano flow electrospray ionization tandem mass spectrometric analysis of peptide samples was carried out using LTQ-Orbitrap Velos (Thermo Scientific, Bremen, Germany) interfaced with Agilent’s 1200 Series nanoflow LC system. The chromatographic capillary columns used were packed with Magic C18 AQ (particle size 5 mm, pore size 100 Å; Michrom Bioresources, Auburn, CA, USA) reversed phase material in 100% ACN at a pressure of 1,000 psi. The peptide sample was enriched using a trap column (75 μm × 2 cm) at a flow rate of 3 ml/min and separated on an analytical column (75 μm × 50 cm) at a flow rate of 350 μl/min. The peptides were eluted using a linear gradient of 7–30% ACN over 65 min. Mass spectrometric analysis was carried out in a data-dependent manner with full scans acquired using the Orbitrap mass analyzer at a mass resolution of 60,000 at 400 m/z. For each MS cycle, 20 most intense precursor ions from a survey scan were selected for MS/MS and fragmentation detected at a mass resolution of 15,000 at m/z 400. The fragmentation was carried out using high-energy collision dissociation (HCD) as the activation method with 40% normalized collision energy. The ions selected for fragmentation were excluded for 30 s. The automatic gain control for full FT MS was set to 1 million ions and for FT MS/MS was set to 0.1 million ions with a maximum time of accumulation of 500 ms, respectively. For accurate mass measurements, the lock mass option was enabled.

### Protein Identification and Quantitation

Identification and quantification were carried out using Max Quant (21) v1.6.17.0 using *Homo sapiens* proteome UP000005640, containing 75776 proteins, from Uniprot (https://www.uniprot.org/) as database for search. Search parameters included trypsin as the proteolytic enzyme with one missed cleavage allowed, oxidation of methionine as a dynamic modification, and alkylation at cysteine as a static modification. Precursor and fragment mass tolerance were set to 20 ppm and 0.1 Da, respectively. The false discovery rate (FDR) was calculated by using a decoy database, and high confidence peptide identifications were obtained by setting a target FDR threshold of 1% at the peptide level (22). Metabo Analyst version (https://www.metaboanalyst.ca/) 5.0 was used to perform pLSDA to analyze the reproducibility between the replicate runs of the samples (23).

The proteins identified in the exosomes were compared to those reported previously in Vesiclepedia (http://microvesicles.org/) (24), an extracellular vesicles database using Fun Rich software v3.1.3 (25). Gene Ontology analysis of all the identified proteins was performed using g: Profiler online tool (https://biit.cs.ut.ee/gprofiler/gost) to identify the biological processes, molecular functions, and cellular components significantly enriched with a *p*-value <0.05 (26). Functional analysis of the identified proteins was performed using Clue GO v2.5.7 application (27) in Cytoscape v3.8.2 (28). Relative quantitation of proteins was carried out in MaxQuant based on the relative intensities of reporter ions released during MS/MS fragmentation of peptides. Only unique and razor peptides for each protein identified were used for quantification, and the proteins that showed more than 1.3-fold change in abundance in glioma samples as compared to control, with a *p*-value <0.05 by ANOVA, were considered as differentially abundant. Probable regulatory factors involved were identified using iRegulon v1.3 application (29) in Cytoscape v3.8.2. Protein-protein interaction map for all the differentially abundant proteins were obtained using STRING (Search Tool for Retrieval of Interacting Genes/Proteins) online tool, version 11 (https://string-db.org/) (30), and visualized using Cytoscape v3.8.2.

### Verification of Galectin-3 BP in Plasma-Derived EVs, Plasma of Individual Glioma Patients by ELISA

Plasma levels of human galectin-3 binding protein (LGALS3BP) were measured in individual glioma, grade I (n=12), grade II (n=20), and grade III (n=8) or control plasma samples (n=40) using ELISA quantitation kit (Thermo Fisher SCIENTIFIC, USA). Fold changes in log2 transformed ratio for galectin-3 binding protein were represented using graph plot.

### Statistical Analysis

Statistical analysis was performed by the Statistical Package for the Social Sciences (SPSS) program for Windows, version 17.0 (SPSS, Chicago, IL, USA). Continuous variables are presented as mean ± SD, median (IQR), and categorical variables are presented as absolute numbers and percentage. Data were checked for normality before statistical analysis. The association between survival status (live and dead) and their grades in glioma patients was performed using the chi square test. Non-normal distribution continuous variable CD63 expression was compared using Mann Whitney U test between glioma patients and controls. A receiver operating characteristics (ROC) analysis was calculated to determine optimal cutoff value for glioma EVs galectin-3 BP protein concentration ng/ml and plasma galectin-3 BP concentration ng/ml of protein. The area under curve, sensitivity, specificity, PPV, and NPV were calculated to analyze the diagnostic accuracy of two concentrations (glioma EVs galectin-3 BP and plasma galectin-3 BP) in predicting glioma patients as compared to controls (healthy individuals). For all statistical tests, value less than 0.05 was considered to indicate a significant difference.

## Results

### Clinical Samples

Glioma grade I (n=12) was 30%, II (n=20) was 50%, and III (n=8) was 20%, respectively. The surgical resection GTR (Guided tissue regeneration): >95% was 37 (92.5%) and STR: >85% was 3 (7.5%) from different grades of glioma patients. Among all the patients, parameters such as seizures were found in 27 (67.5%), vomiting in 20 (50%), vision problem in 25 (62.5%), and memory loss in 14 (35%). Second surgery was performed in 9 (22.5%) patients, and survival rate was 31 (77.5%) as shown in (**Table 1**).

**Table 1:** Clinicopathologic characteristics of collected glioma patients.

Glioma		No. of patients N = 40	%
**Glioma grades**	**I**	12	30.0
	**II**	20	50.0
	**III**	8	20.0
**Extent of surgical resection**	**(GTR: >95%)**	37	92.5
	**STR: > 85–95%**	3	7.5
**Seizures**		15	37.5
**Headache**		27	67.5
**Vomiting**		20	50.0
**Vision problem**		25	62.5
**Memory loss**		14	35.0
**Second surgery**		9	22.5
**Survival**		31	77.5

Glioma patients’ survival was found 12 (100%) in grade I, 15 (75.0%) in grade II, 4 (50.0%) in grade III. And death of the glioma patients found in grade I was 0 (0%), grade II was 5 (25.0%), grade III was 4 (50.0%), respectively. The survival rate of the patients is reducing as the grades of glioma increases, which was found statistically significant p=0.030 as shown in (**Supplementary Table 1**).

### Isolation and Physical Characterization of Glioma-Derived Extracellular Vesicles

To verify the potential value of circulating extracellular vesicles as biomarkers for gliomas, extracellular vesicles were isolated from plasma of patients with different grades of glioma (grade I, II, and III) at diagnosis (before surgery) and, in parallel, from age- and sex-matched healthy controls by a combination of filtration and ultracentrifugation. Further NTA analysis revealed that particle sizes were well within the reported range of circulating EVs (mode particle size of groups 35–181 nm), with no significant differences between groups. Western blot analysis of plasma-derived exosomal proteins profiling of CD63 exosomal marker was visualized in different grades of glioma as depicted (**Supplementary Figure S1**). These experiments were repeated three times for each grade

### Flow Cytometric Analysis of Tetraspanin CD63 Expression in Different Grades of Glioma

We performed a flow cytometric analysis of glioma-derived extracellular vesicles, and it shows the results of a representative experiment out of the three performed with similar results. The flow cytometric assay revealed good levels of signals for the tetraspanin molecule CD63 tested as exosomal marker on vesicles derived from different-grade glioma patients (grade I, II, III), and the same proteins were detected by flow cytometry in control sample (healthy individuals). The expression of exosomal marker CD63 was increased in grade I>grade II>grade III, and *p*-value was highly significant as shown in **Supplementary Table 2**.

### Circulating Extracellular Vesicles Carry a Wide Variety of Functional Proteins

**Figure 2:**
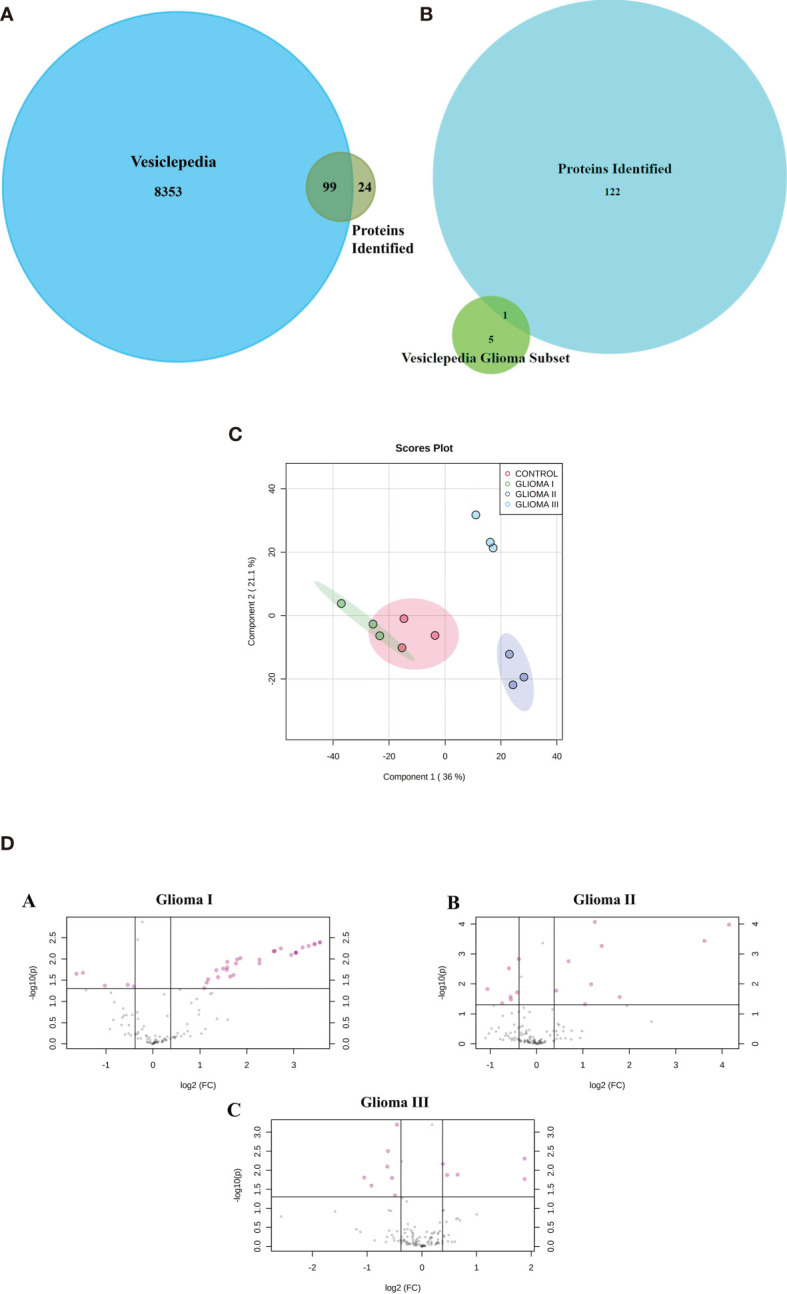
Proteomic analysis using iTRAQ labelling. **(A)** Venn diagram indicating the protein identified in our study and those reported in Vesiclepedia. **(B)** Venn diagram indicating proteins identified in the present study along with the previously reported in Vesiclepedia from exosomes in glioma. **(C)** pLSDA analysis of replicate runs of each pooled sample. **(D)** (A–C) Differential expression Analysis volcano plots of log2 (fold change) VS log10 (p value) indicates the proteins (colored in pink) showing statistically significant difference abundance in the different grades of glioma as compared to control.

**Figure 3:**
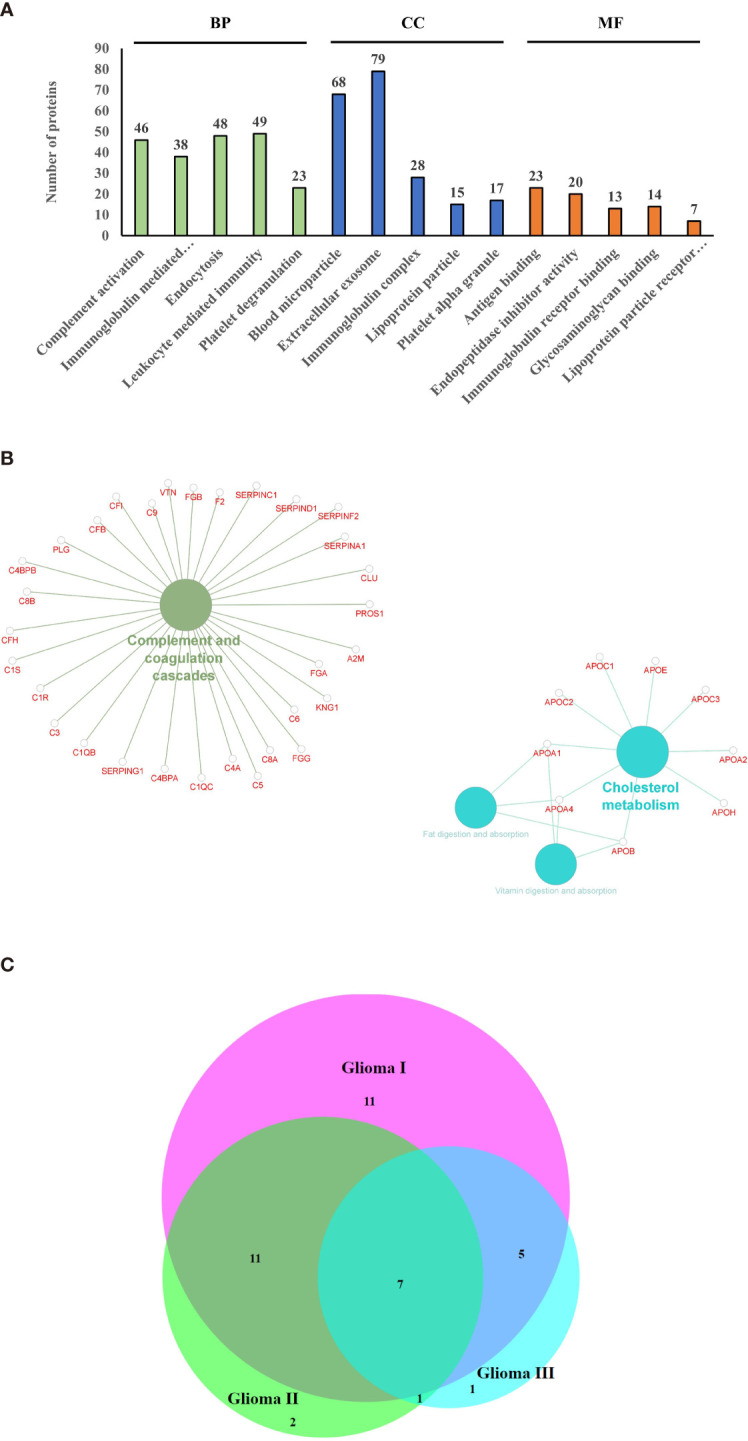
Gene ontology and pathway analysis. **(A)** Gene ontology terms that were found to be enriched after functional annotation of identified protein using g-profiler and the number of proteins under each category (BP, Biological Process; CC, Cellular Component; MF, Molecular Function). **(B)** Pathways that were found to be enriched among the identified proteins. **(C)** Venn diagram indicating the common differentially expressed proteins between the grades.

Using mass spectrometry-based proteomics, we could identify 123 proteins from the extracellular vesicles, out of which 99 have been previously reported to be present in exosomes when compared with the Vesiclepedia database (**Figure 2A**). However, only one protein from our study, fibronectin, has been identified earlier in exosomes in glioma (**Figure 2B**), implying that we were able to identify over a hundred novel proteins, which were not previously reported to be associated with exosomes in glioma. The pLSDA of all the acquisitions showed good reproducibility among all the replicate runs of samples (**Figure 2C**). Differential expression in the different grades of glioma as compared to healthy control shows statistically significant as depicted (**Figure 2D**). As expected for extracellular vesicles, a large number of proteins were annotated as extracellular exosome components in the GO cellular component (CC) analysis of the identified proteins using g: profiler online tool (**Figure 3A**). Other enriched CC terms included blood microparticle, immunoglobulin complex, lipoprotein particle, and platelet alpha granules. Proteins having molecular function (MF) of antigen binding, endopeptidase inhibition, and glycosaminoglycan binding were also found to be enriched in the GO analysis. Interestingly, biological processes (BP) like complement activation, immunoglobulin-mediated immunity, and leukocyte-mediated immunity were found to be enriched. Additionally, KEGG pathway analysis by Clue GO also detected that many proteins were involved in complement activation (**Figure 3B**). Since interaction of the complement system with tumor cells has been previously implicated in enhanced tumor growth and cancer metastasis (31–33), the presence of proteins of the complement system in the extracellular vesicles may indicate the activation of this system in glioma patients. Another pathway that was enriched in Clue GO analysis was cholesterol metabolism, but this may not be a result of glioma pathology but instead be a result of co-isolation of lipoprotein particles with extracellular vesicles during the purification process (34). This is also supported by the fact that we have identified many components of lipoproteins like Apo A1, Apo C1, and Apo E in our study However, a recent review by Ahmed et al. has discussed the potential of cholesterol metabolism as a therapeutic target for glioblastoma, and further studies in this direction may indicate the role of this pathway in GBM (35).

### Circulating Extracellular Vesicle Proteins Distinguish Patients With Glioma From Healthy Controls

A number of proteins showed altered expression in the circulating extracellular vesicles from patients with glioma as compared to those from control subjects (**Supplementary Figures S1A–C**). Analysis by Max Quant identified 34, 21, and 14 proteins to be differentially abundant by more than 1.3-fold in the three grades of glioma, respectively, in comparison with the control samples, out of which, seven proteins were found to be differentially abundant in all three grades (**Figure 3C**), with galectin-3 binding protein, C-reactive protein (CRP), and Serum amyloid A2 (SAA2) being upregulated across all grades. Only the proteins that showed *p*-value < 0.05 by ANOVA were considered as differentially expressed. Analysis by iRegulon predicted YY1 (Yin Yang 1) and STAT1 (Signal Transducer and Activator of Transcription) to be possible regulators of a number of proteins that are upregulated in the early grades of glioma (**Supplementary Figures S2A, B**). YY1 or Yin Yang 1 transcription factor is known to regulate a number of proteins associated with cell division, differentiation, and DNA repair; and a number of evidences indicate its role in the progression of glioma (36). Conversely, STAT1 or Signal Transducer and Activator of Transcription controls the expression of different proteins involved in cell viability, and its activation has been previously reported to be involved in suppression of glioma carcinogenesis (37–39).

### Identification of Galectin-3 Binding Protein Interaction Map by Targeted Analysis of Glioma Patient EVs

**Figure 4:**
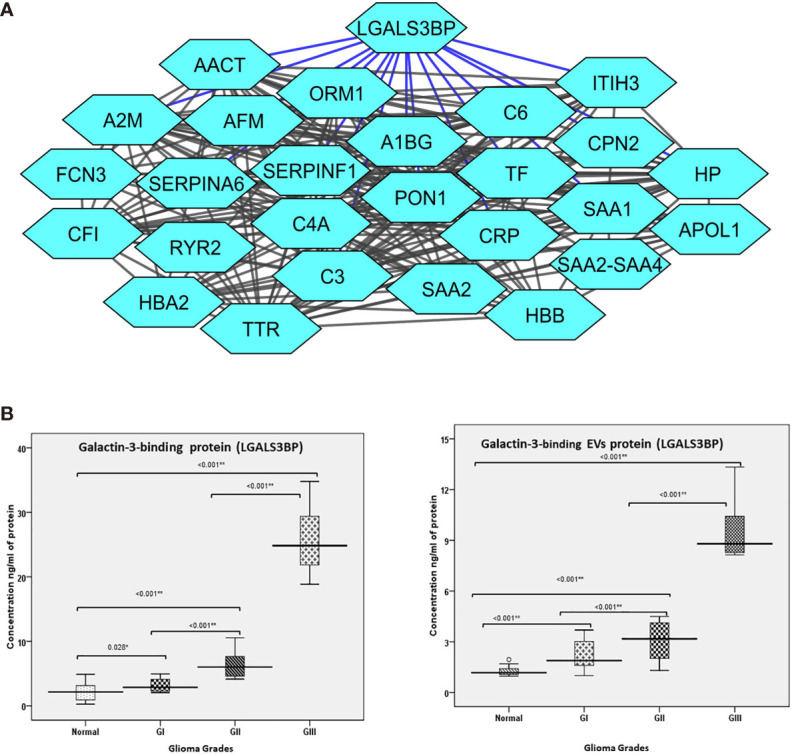
**(A)** Protein interaction map of galectin-3 binding protein (LGALS3BP) and its interacting proteins and their fold change observed in different grades of glioma. The blue lines represent direct interaction with galectin-3 binding protein. **(B)** Error bar represents the range between different grades of glioma and normal healthy control of the collected patients in the verification cohort using ELISA technique. **(B)** (a) Error bar represents galectin-3 binding protein (LGALS3BP) in plasma. **(B)** (b) Error bar represents galectin-3 binding protein (LGALS3BP) in extracellular vesicle (EV) protein. * = Significant and ** = highly significant.

An interesting finding of the proteomic analysis was that galectin binding protein (LGALS3BP) was seen to be upregulated across different grades of glioma. Also, protein-protein interaction map of the differentially expressed proteins indicated a number of proteins to be interacting directly with LGALS3BP, including CRP, (Complement component) C6, SERPINF1 (Serpin Family F Member 1), and CPN2 (Carboxypeptidase N Subunit 2) (**Figure 4A**). LGALS3BP, also known as Mac-2 binding protein (Mac-2BP) or tumor-associated antigen 90K, is a secreted glycoprotein that has been widely studied as a marker for cancer progression and metastasis (40). It is a ligand for Galectin-3, a lectin that binds to β-galactoside sugars and is involved in cell-cell and cell-matrix adhesion. Although there are a number of studies reported that suggest the involvement of Galectin-3 in gliomas (41), the LGALS3BP is not widely studied yet. However, our approach through this set of experiment is to validate its expression in plasma-derived extracellular vesicles as well as in whole crude plasma samples for larger number of glioma patients using the technique called ELISA and further evaluate the results to describe its role as an early marker for glioma.

### Clinical Quantification of LGALS3BP in Plasma-Derived EVs and Plasma From Glioma

As differential abundance of the functionally relevant protein, Galectin-3 BP, was detected in the different grades in mass spectrometry results, this protein was selected for clinical verification in both plasma-derived EVs and blood-plasma by ELISA.

In this approach, we compared the plasma specimens as well as plasma-derived EV levels from patients diagnosed with glioma (Gr I, II, III) samples (n=40) derived from matched healthy individuals (n=40). We observed significantly elevated level of Galectin-3 BP in individual plasma specimens from glioma (Gr I, II, III) patients as compared with healthy individuals. The fold changes of this protein, as log2 transformed ratio, are shown in the graph plot (**Figures 4B** **, C**).

### Glioma Galectin-3 BP to Predict Mortality in Glioma Patients

Receiver operating characteristics (ROC) analysis shows the cutoff value of >=2.015 found to predict mortality of the glioma patients along with sensitivity 77.8%, specificity 35.5%, PPV 25.9%, NPV 84.6%. The accuracy of Glioma Galectin-3 BP EVs protein concentration ng/ml was 45.0%, respectively. Galectin-3 BP threshold value to predict the mortality rate in glioma patients is depicted in (**Table 2**).

**Table 2:** Correlation of plasma-derived EV concentration and plasma protein concentration with galectin-3 BP.

Galectin 3 BP in EVs and plasma	Threshold value	AUC (95% CI)	Sensitivity	Specificity	PPV	NPV	Accuracy
Glioma EVs protein concentration ng/ml	>=2.015	0.677 (0.486–0.869)	77.8%	35.5%	25.9%	84.6%	45.0%
Plasma concentration ng/ml of protein	>=7.656	0.767 (0.600–0.934)	66.7%	77.4%	46.2%	88.9%	75.0%
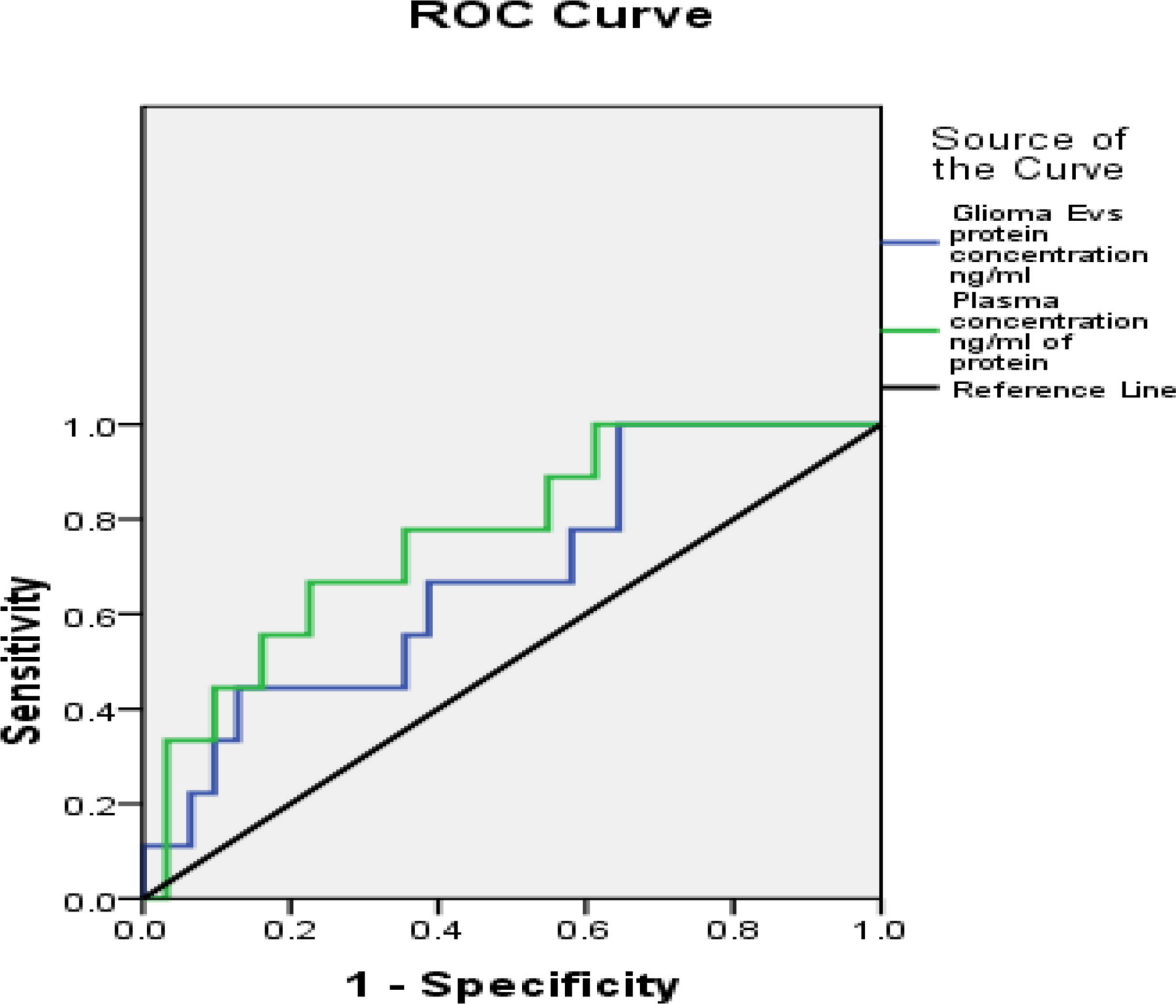

Receiver operating characteristics (ROC) analysis of glioma plasma (n=40). Threshold were determined as the points with minimum distance from 100% specificity in the ROC curve for glioma plasma-derived EVs (n=40) and plasma of glioma patient (n=40).

## Discussion

A major clinical obstacle in cancer is the evaluation of molecular and cellular changes in tumor pathogenesis during cancer progression. Glioma patients experience local recurrence of the tumor despite aggressive trimodal therapy that includes surgery, chemotherapy, and radiotherapy (42). While imaging technologies are emerging, they are costly and often not applicable for frequent use and show the side effects. There is often a debate regarding the proper assessment of magnetic resonance imaging (MRI) in the routine surveillance of patients with glioma. There are challenges in the neuroimaging evaluation of patients undergoing treatment including variability in image acquisition parameters, inter-rate measurement variability, difficulty in measurement of irregularly shaped tumors, and inconsistent interpretation of treatment-related radiographic changes (43–45). Nowadays circulating tumor cells (CTCs) and circulating tumor DNA (ctDNA) are the most frequent analytes of liquid biopsy assays (46–48). EVs hold great engrossment over the last decade, which is owing to the belief that EVs could possibly play a strong role in tumor pathology. In the present study, various techniques were performed to characterize the extracellular vesicles; also, the expression of galectin-3 binding protein derived from plasma EVs was found to be strikingly high in patients having various grades of glioma as compared to healthy individuals. These results were obtained through proteomic analysis. It is already known that Galectin-3 increases proliferation of breast, prostate, pancreatic, and tongue tumors. This might be due to the interaction of galectin-3 with the APC/Axin/β-catenin complex in the nucleus. The members of the Bcl-2 family are probably not the only galectin-binding partners implicated in apoptosis regulation. Synexin, a calcium and phospholipid-binding protein, has been shown to drive the perinuclear translocation of galectin-3, which is essential to its anti-apoptotic function. Galectin-3 also increases vascularization of prostate tumors and melanomas. The major three homeostatic roles of galectins include their ability to make tumor cells more adaptive to stressed conditions. They may affect detachment and migration of tumor cells after secretion and also affect angiogenesis. Galectins (multimeric in the case of galectin-3) induce cross-linking of the receptors and formation of a lattice that triggers a cascade of transmembrane signaling events. This protein is found mainly in cytoplasm and is also detected in nucleus, cell surface, extracellular space, and mitochondria (49–51). It helps in pre-mRNA splicing and provides protection against apoptosis. It is also located on cell surface and extracellular space depicting its role in cell-cell and cell-matrix adhesion. It also takes part in cell activation, differentiation, cell cycle, and has been seen to be linked with various diseases like cancer, inflammation, fibrosis, stroke, and heart attack (49, 52–54).

Several studies have been carried out on extracellular vesicles from different malignancies such as melanoma (55), prostate cancer (56), bladder cancer (57), colorectal cancer (58), and others. Due to the abundant expression of tumor-associated antigens, tumor-derived extracellular vesicles have been considered as source of antigenic determinants to be exploited in the design of cancer vaccine strategies (59, 60). To the best of our knowledge, this is the first report of galectin-3 binding protein as a plasma-based marker for glioma. Mass spectrometry-based proteomics provides a useful tool for biomarker discovery since it can be used to quantify a large number of proteins simultaneously across many samples. There have been many previous studies using proteomics in glioma that have tried to identify associated biomarkers mainly using tumor biopsy samples (61) or cerebrospinal fluid from patients (62). Niclou et al. and Kalinina et al. have provided a comprehensive review of the different proteomic studies conducted for glioma and the different samples used (63, 64). However, both tumor biopsy and CSF collection are invasive, and a comparatively less invasive source such as plasma or serum may prove to be more useful at clinical level. Recently, plasma exosomes are emerging as a rich source of biomarkers for many diseases; and many studies on exosomes from different cancers such as breast (65, 66), bladder (67), and ovarian (68) have led to identification of proteomic signatures of these cancers. A recent review by Simona et al. neatly describes the contribution of mass spectrometry-based proteomics of tumor-derived exosomes in discovery of biomarkers in different cancer (69).

In case of glioma, many *in vitro* studies have predicted the importance of exosomes using cell culture (70, 71), as well as animal models (72). There have been few proteomic studies on glioblastoma, “the most malignant form of glioma”, based on extracellular vesicles (73). The major part from previous studies was not inclined towards EVs based comparative analysis of different grades of glioma that could possibly provide biomarkers for early detection of these particular brain tumors. We have tried to address this issue in our study using pooled extracellular vesicles from patients with different grades of glioma and used high-resolution quantitative mass spectrometry to identify proteins showing differential abundances across these grades. Our approach was highly effective since we were able to identify over a hundred proteins that were not previously reported to be associated with exosomes in glioma. Particularly of interest is galectin-3 binding protein, which we found to be upregulated in different grades of glioma and has also been reported in The Human Protein Atlas v15 (www.proteinatlas.org) to be localized in vesicles of glioblastoma cell lines and overexpressed in glioma tissue (74). We were able to validate its overexpression in individual plasma exosome samples from glioma patients as well. Therefore, it has a potential to be a biomarker for early detection of glioma, and further studies are needed to be performed in a larger cohort of samples to validate its application. Our study is a pilot study towards the development of diagnostic methods using galectin-3 binding protein as a plasma-based marker for the early detection of glioma.

## Conclusion

This integrated strategy would provide a high confidence method of EVs-derived, tumor-related circulatory molecules, developing assay for early detection of glioma and also provides the information of progression in tumor grades. The present research provides a galectin-3 binding protein (LGALS3BP) as novel biomarker for early detection of glioma (brain tumor).

## Data Availability Statement

The datasets presented in this study can be found in online repositories. The names of the repository/repositories and accession number(s) can be found in the article/**Supplementary Material**.

## Ethics Statement

The studies involving human participants were reviewed and approved by Sir Ganga Ram Hospital, Human Ethical Committee (Ref no. EC/10/17/1270), Delhi, India. The patients/participants provided their written informed consent to participate in this study.

## Author Contributions

RR executed the idea and drafted the manuscript. KC has performed the experiments. NTA analysis was done at the department of pathology, AIIMS, India. PG helped to edit manuscript. Control blood samples were provided by Department of Blood Transfusion, SGRH. MK and RB have done bioinformatics analysis. SC, RA, SK, and AG have provided the samples and clinical data. SJ provided the IHC findings. Statistical analysis was done by PC. NG gives final input in the manuscript. All authors contributed to the article and approved the submitted version.

## Conflict of Interest

The authors declare that the research was conducted in the absence of any commercial or financial relationships that could be construed as a potential conflict of interest.

## Publisher’s Note

All claims expressed in this article are solely those of the authors and do not necessarily represent those of their affiliated organizations, or those of the publisher, the editors and the reviewers. Any product that may be evaluated in this article, or claim that may be made by its manufacturer, is not guaranteed or endorsed by the publisher.

## Supplementary Material

Supplementary Figure S1 Isolation and Characterization of Extracellular vesicles from human blood plasma of glioma patients. A, Nanoparticle Tracking Analysis (NTA) of plasma derived EVs. B, Cd63 marker detection by western blotting. C, Flow Cytometry Analysis.

Supplementary Figure S2 Proteomic analysis using iTRAQ. A and B, iRegulon based prediction of transcription factors identified YY1 and STAT1 as regulators of a number of proteins that are up-regulated in the early stages of glioma.

Supplementary Table 1 Survival status of the collected glioma patients.

Supplementary Table 2 CD63 expression in different grades of glioma (I, II, III and controls).

Supplementary Data Excel sheet of LC-MS/MS data.
