# Supplementary figures and images for: Plasma-Derived Extracellular Vesicles Reveal Galectin-3 Binding Protein as Potential Biomarker for Early Detection of Glioma
Source: Front Oncol. 2021 Nov 26;11:778754. doi: 10.3389/fonc.2021.778754 (PMC8661035; doi:10.3389/fonc.2021.778754)

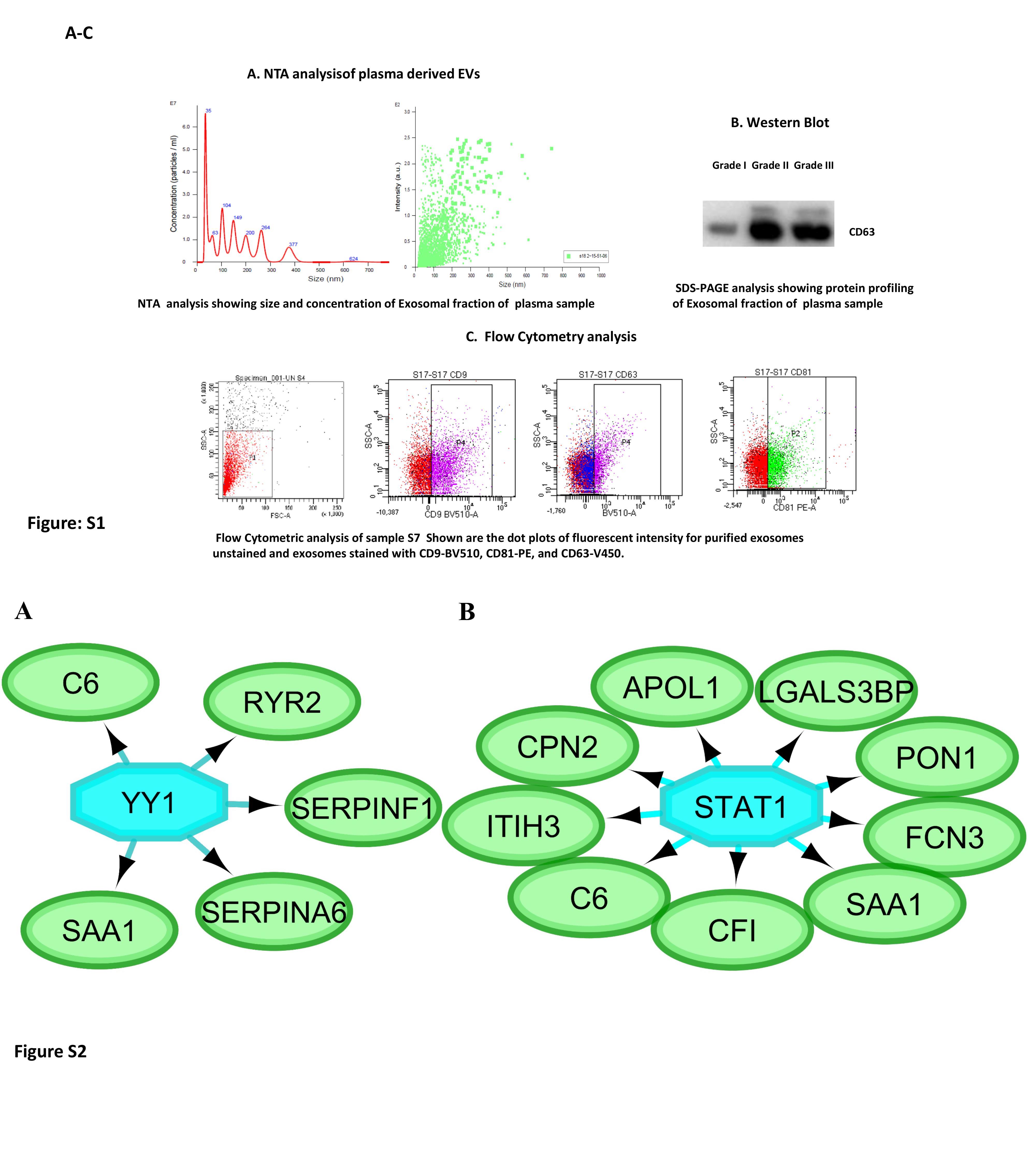

Supplement: Supplementary Figure S1 — Isolation and Characterization of Extracellular vesicles from human blood plasma of glioma patients. A, Nanoparticle Tracking Analysis (NTA) of plasma derived EVs. B, Cd63 marker detection by western blotting. C, Flow Cytometry Analysis. [file Image_1.jpeg]
