# Supplementary material for: Plasma-Derived Extracellular Vesicles Reveal Galectin-3 Binding Protein as Potential Biomarker for Early Detection of Glioma
Source: Front Oncol. 2021 Nov 26;11:778754. doi: 10.3389/fonc.2021.778754 (PMC8661035; doi:10.3389/fonc.2021.778754)
Supplement: Supplementary Table 1 — Survival status of the collected glioma patients. [file Table_1.doc]

**Supplementary Table 1:** Survival status in glioma patients’ %

| **Survival Status** | **Glioma Grades** | | | **p value** |
| --- | --- | --- | --- | --- |
|  | **Grade I** | **Grade II** | **Grade III** |  |
|  | **Frequency (%)** | **Frequency (%)** | **Frequency (%)** |  |
| Dead | 0 (0.0%) | 5 (25.0%) | 4 (50.0%) | 0.030 |
| Live | 12 (100%) | 15 (75.0%) | 4 (50.0%) |  |
| Total | 12 (100%) | 20 (100%) | 8 (100%) |  |

**Supplementary Table 2:** CD63 expression in different grades of glioma (grade I, II, III) and controls

| **EVs marker** | **Cases (n=40)** | | **Controls (n=40)** | | **p value** |
| --- | --- | --- | --- | --- | --- |
|  | **Mean ± SD** | **Median (IQR)** | **Mean ± SD** | **Median (IQR)** |  |
| **CD 63** | 0.34 ± 0.24 | 0.23 (0.20 - 0.31) | 0.16 ± 0.05 | 0.14 (0.12 - 0.21) | <0.001 |
